# The natural history of HIV infection

**DOI:** 10.1097/COH.0b013e328361fa66

**Published:** 2013-06-06

**Authors:** Caroline A. Sabin, Jens D. Lundgren

**Affiliations:** a Research Department of Infection and Population Health, University College London (UCL), Royal Free Campus, London, UK; b Department of Infectious Diseases, Copenhagen University Hospital/Rigshospitalet and Copenhagen HIV Programme, University of Copenhagen, Copenhagen, Denmark

**Keywords:** antiretroviral treatment, disease progression, elite control, long-term nonprogression, natural history, primary HIV infection

## Abstract

**Purpose of review:**

To review recent published literature around three areas: long-term nonprogression/viral control; predictors of viral load set point/disease progression; and the potential impact of antiretroviral therapy (ART) in early HIV infection.

**Recent findings:**

The natural course of untreated HIV infection varies widely with some HIV-positive individuals able to maintain high CD4 cell counts and/or suppressed viral load in the absence of ART. Although similar, the underlying mechanistic processes leading to long-term nonprogression and viral control are likely to differ. Concerted ongoing research efforts will hopefully identify host factors that are causally related to these phenotypes, thus providing opportunities for the development of novel treatment or preventive strategies. Although there is increasing evidence that initiation of ART during primary infection may prevent the immunological deterioration which would otherwise be seen in untreated HIV infection, recent studies do not address the longer term clinical benefits of ART at this very early stage.

**Summary:**

A better understanding of the relative influences of viral, host, and environmental factors on the natural course of HIV infection has the potential to identify novel targets for intervention to prevent and treat HIV-infected persons.

## INTRODUCTION

In the early days of the HIV epidemic, knowledge about the natural history of HIV accrued rapidly. However, the widespread use of effective antiretroviral therapy (ART) brought a shift in focus of the research community away from studies of natural history to those of treated infection. Nevertheless, recent years have seen many advances in our knowledge about natural history. For the purposes of this review, we will focus on three areas of relevance to treating clinicians: long-term nonprogression and viral control; predictors of viral load set point and disease progression; and the potential impact of ART in early HIV infection.

## LONG-TERM NONPROGRESSORS AND ELITE CONTROLLERS

The natural course of untreated HIV infection varies widely. The past decade has seen considerable interest in the identification of subgroups of HIV-positive persons who exhibit distinct patterns of disease progression. It is hoped that the information obtained through the identification of such individuals might provide insight for the development of vaccines and novel treatment approaches.

Long-term nonprogressors (LTNP) are individuals who remain asymptomatic for a prolonged period of time off ART with a high CD4 cell count (see reviews by Poropatich and Sullivan and Gaardbo *et al.*[1,2^▪^]). Although it is widely reported that 1–5% of the HIV-positive population are LTNP, these estimates are complicated by the fact that there is no standardized definition of a LTNP, and thus definitions used (and the way in which they are applied, particularly in the presence of varying follow-up and irregularly measured CD4 cell counts) differ widely (Table 1) [3–5,6^▪▪^,^7^,^8^]. For example, Madec *et al.*[3] identified asymptomatic individuals who remained off ART for more than 8 years with a CD4 cell count more than 500 cells/μl; using this definition, 9% of their clinic population were identified as LTNP. Using a similar definition but with only 7 years of follow-up, Okulicz *et al.*[4] reported a prevalence of 5.02% in a military cohort. In contrast, only 0.4% of patients in the French Hospital's Database on HIV were identified as LTNP [5]. In a UK study, Mandalia *et al.*[6^▪▪^] identified ART-naive asymptomatic individuals infected with HIV for more than 7 years. Of 312 such patients, only 50 had stable CD4 cell counts, with only 13 having CD4 cell counts consistently in the normal range. Thus, LTNP represented only 0.2% of patients attending for care, a far lower rate than that reported by Okulicz *et al.*, presumably because of the additional requirement that individuals had stable CD4 cell counts.

**Box 1:**
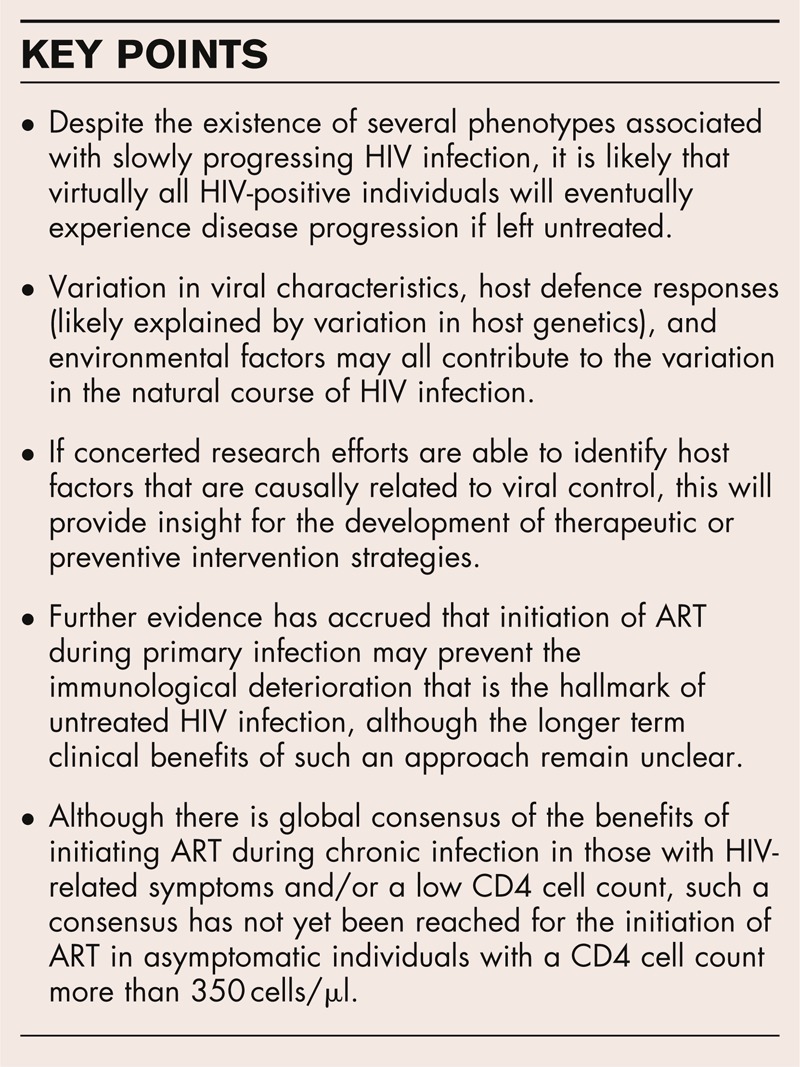
no caption available

LTNP status can be lost, and thus the reported prevalence of LTNP within a study will depend on the required period of follow-up. In Madec's study [3], LTNP status was lost after 8 years in 36 of the 60 LTNP; loss of LTNP status was generally because of declining CD4 cell counts and initiation of ART, although a small number of individuals experienced Centers for Disease Control stage B/C disease. Predictors of loss of LTNP status were a high baseline HIV DNA level and a more rapid increase in HIV DNA over the first years of follow-up, suggesting the presence of ongoing (but low-grade) viral replication. Indeed, HIV RNA levels in plasma increased by 0.04 log_10_ copies/ml per year over the first 8 years after diagnosis. When the required period of follow-up was increased from 7 to 10 years in a military cohort [4], the prevalence of LTNP status dropped from 5 to 2%. The fact that an individual's LTNP status can change has led some to suggest that rather than representing a distinct group of HIV-positive individuals, LTNP are more likely to represent individuals at one tail end of a Normal distribution [6^▪▪^]. As such, it is likely that virtually all HIV-positive persons will eventually experience disease progression if left untreated.

More recently, interest has shifted towards the identification of individuals who are able to suppress HIV replication to such an extent that viral load levels remain undetectable in the absence of ART [9]. These individuals are generally referred to as elite controllers or viral controllers. In the military cohort described by Okulicz *et al.*[4] elite controllers were defined as ART-naive patients infected with HIV for more than 12 months with at least three longitudinal undetectable HIV RNA determinations. Individuals were allowed to have occasional HIV RNA levels up to 1000 copies/ml as long as these episodes represented the minority of all determinations. These elite controllers were distinguished from viremic controllers in whom the majority of viral loads were in the range 1000–2000 copies/ml. In total, 0.6% of 4586 individuals were identified as elite controllers and 3.3% as viremic controllers. Virological control was established a median of 1 year after seroconversion, lasted for 846 and 1085 days in elite controllers and viremic controllers, respectively, and was associated with a reduced risk of clinical progression. Interestingly, although elite controllers experienced an initial CD4 cell count increase followed by stabilization, viremic controllers generally experienced a loss of CD4 cells. Goujard *et al.*[10] confirmed that elite controllers status is established early after primary infection in the Agence Nationale de Recherche sur le Sida PRIMO cohort.

Although there is clearly overlap between the LTNP and elite controller groups, not all LTNP have a suppressed viral load, and not all elite controllers have high CD4 cell counts. Furthermore, LTNP status is not necessarily protective against clinical progression. An early study from the CASCADE group [11] suggested that 15 and 7% of elite controllers infected with HIV for more than 16 years had CD4 cell counts less than 350 cells/μl or AIDS, respectively. A more recent study from the group [12] demonstrated that the proportion of elite controllers with at least one CD4 cell count less than 500 cells/μl ranged from 45 to 53%, depending on the definition of elite controllers. Sedaghat *et al.*[13] noted that CD4 slopes in elite controllers varied substantially with rates of CD4 loss of up to 53 cells/μl per year in some individuals. Using a highly sensitive viral load assay, Pereyra *et al.*[14] reported that the median viral load was 2 copies/ml in 90 elite controllers. Low-level viremia was present in the majority of elite controllers; CD4 loss was more common among those with low-level viremia than in those without detectable virus. Boufassa *et al.*[15] reported that clinical and immunological progression in elite controllers was restricted to those experiencing viral load ‘blips’.

More recently, Groves *et al.*[16^▪▪^] identified ART-naive patients who had maintained a viral load less than 2000 copies/ml for more than 12 months. Typical controllers had an average recent CD4 cell count more than 450 cells/μl (2.1% of population), whereas discord controllers had an average recent CD4 cell count less than 450 cells/μl (0.6%). Thus, in this study, the term discord controller was used to identify individuals who had experienced a loss of CD4 cells despite viral control. There were no significant differences in viral load or demographic factors between the two groups. Interestingly, there was a suggestion of a higher frequency of infection with subtype C virus in discord controllers (40% of patients) compared with the entire clinic population of whom 25.1% were infected with subtype C; whether this overrepresentation relates to specific features of subtype C virus itself, or whether subtype C is merely a marker of infection in certain regions of the world with specific host genetic and environmental factors is, however, unclear.

As with LTNP, several studies have attempted to identify factors associated with elite controller status. Yang *et al.*[17] considered the relative and absolute numbers of naive T-cells in a cohort of elite controllers with normal or declining CD4 cell counts and in ART-treated individuals. The relative proportions of naive CD4 and CD8 T cells were reduced in elite controllers, resembling the patterns seen in individuals with untreated progressive HIV infection. The authors concluded that loss of naive CD4 T cells is a universal feature of elite controllers, despite the ability of such individuals to maintain undetectable viral loads. Chen *et al.*[18] suggested, based on in-vitro experimentation, that CD4 naive lymphocytes from elite controllers were less susceptible to HIV infection than such lymphocytes from progressors or uninfected individuals. This specific feature was linked with upregulation of a cellular kinase (p21). Mahnke *et al.*[19^▪^] compared patients maintaining low levels of viremia (controllers) with those experiencing disease progression within 2 years of diagnosis (fast progressors) and with progressive disease not requiring ART (slow progressors). Although beneficial human leukocyte antigen (HLA) types (HLA-B∗27, B∗57, and B∗58) were seen more commonly in controllers (57%) they were also expressed by 23% of slow progressors. Progressors were more likely to be coinfected with GB virus C than controllers, coinfection with which has previously been reported by some to be associated with a slower rate of disease progression in HIV infection [20], but the CCR5 Δ32 mutation was similarly distributed across the groups. Plasma HIV viral load did not differ between progressors, but cell-associated viral load was elevated in fast progressors and lowered in controllers. Although the frequency of CD38^+^CD8^+^ T cells was a strong predictor of disease progression in the first year after HIV infection, and was sufficient to distinguish progressors from controllers, this measurement alone could not differentiate between fast and slow progressors.

As the two groups of individuals appear to be clinically distinct, suggesting differences in the processes that lead to long-term nonprogression and elite control, several research groups have attempted to investigate whether there are any demographic or biological differences between these two patient groups [4]. Groves *et al.*[16^▪▪^] reported a more marked depletion of the naive T-cell subset in discord controllers than in typical controllers but a trend towards increased activated effector memory CD4 cells in typical controllers. CD8 T-cell activation was increased to a similar level (compared with noncontrollers) in both controller groups. The authors concluded that despite the lower CD4 cell counts in discord controllers, their CD8 T-cell activation pattern more closely resembled that of typical controllers. As with other studies, the discord controllers had higher viral DNA loads than the typical controllers, suggesting continued viral replication in this subgroup.

Shaw *et al.*[21] compared viral controllers (individuals with viral load <1000 copies/ml for >5 years) to viremic slow progressors (individuals with a viral load >10 000 copies/ml but who had maintained a CD4 cell count >500 cells/μl for >7 years) and viremic progressors (individuals infected for a similar time with viral load >10 000 copies/ml but CD4 cell count <500 cells/μl). Viremic slow progressors had higher levels of markers of mucosal immune activation and low numbers of mucosal Tregs, suggesting that factors other than immune activation account for this phenotype. Gaardbo *et al.*[7] reported that LTNP had a higher frequency of activated CD4 and CD8 cells compared with viral controllers, but similar levels to progressors. Ballana *et al.*[8] confirmed results from other studies [22] showing that a single nucleotide polymorphism 35 kb upstream of the *HLA-C* gene (−35C/T) is associated with LTNP status.

**FIGURE 1:**
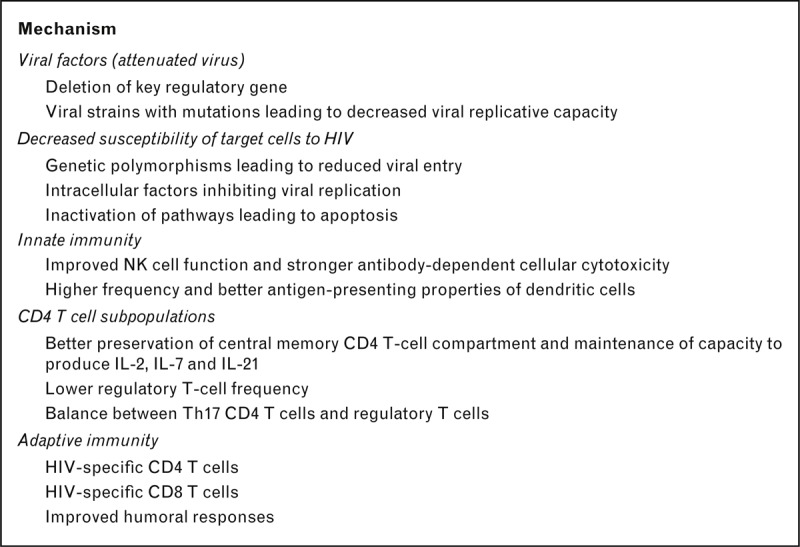
Potential mechanisms of viral suppression in HIV controllers. Adapted from [24].

HIV-specific CD4 activation is a hallmark of viral control [23] but, as outlined above and reviewed recently [24] (see Fig. 1), many other host factors have been linked with this phenotype, including cellular restriction factors such as APOBEC, tetherin, and the recently identified SAMHD1 [25,26]. In addition, several viral factors may also play a role, including deletions or mutations with the viral genes [27] that may have an impact on the ability of the virus to replicate. Concerted ongoing research efforts will hopefully clarify whether any of these host factors are causally related to viral control or merely reflect intrinsic variations in the ability of the virus to replicate. If any host or viral factors do have an important influence on viral replication, such a discovery will open a field of possibilities aimed at enhancing or mimicking these host factors as part of a therapeutic or preventive intervention strategy.

## OTHER PREDICTORS OF VIRAL LOAD SET POINT AND CD4 LOSS

The possibility that there may be a link between the viral load set point and the viral load of the infecting partner was raised by Hecht *et al.*[28] who demonstrated that in 24 transmission pairs, the viral load in the donor was closely associated with the viral load at presentation in the seroconverting partner (correlation coefficient = 0.55). Using a novel phylogenetic approach to determine heritability, Alizon *et al.*[29] concluded that up to half the variance in the viral load set point among individuals in the Swiss HIV Cohort Study could be heritable from their infecting partners. These observations support the notion that HIV has varying intrinsic replicative capacity and suggest that this feature is maintained after transmission.

Predictors of the viral load set point were investigated by Lingappa *et al.*[30^▪▪^] in 141 African seroconverters. In multivariable analysis, higher viral loads in the source partners were associated with higher viral load set points in the seroconverters. The proportion of variation in set point that could be attributed to the viral load of the source partner, after controlling for other factors, was 6%. Despite this low proportion, the authors concluded that the source partner viral load was the most significant predictor of the viral load set point in the seroconverter. Yue *et al.*[31^▪▪^] also noted the relatively small proportion of variance in the viral load set point that could be explained by the viral load in the source partner. In an analysis of 195 transmission pairs from Zambia, the viral load in source partners explained only around 2% of the variance in viral load set points of seroconverters. Overall, the viral load set point was a function of the source partner viral load, the sex of the seroconverter, the HLA class I alleles of the seroconverter, and the sharing of HLA-I alleles between partners in a transmission pair. Together, these factors accounted for up to 37% of variance in the viral load set point. Roberts *et al.*[32] reported that the concentration of five plasma cytokines (IL-12p40, IL-12p70, IFN-γ, IL-7, and IL-15) predicted 66% of the variation in viral load set point in 40 South African women. Grinsztejn *et al.*[33^▪^] reported that women in the Prospective Evaluation of Antiretrovirals in Resource-Limited Settings (PEARLS) study had a lower mean preART viral load than men; whereas the sex difference was related to the CD4 cell count, it was independent of country and persisted in those with a CD4 cell count less than 200 cells/μl.

Other predictors of disease progression include transmission of resistant strains of HIV [34] and the envelope diversity of the virus in the individual after seroconversion [35^▪^]. In the latter study, viral diversity at 1-year postseroconversion was associated with accelerated progression to clinical AIDS or a low CD4 cell count, although not with the viral load set point itself. The authors could not determine whether viral diversity is a direct cause of immunodeficiency, or a consequence of the individual's response to infection. In a small study of 50, chronically infected, asymptomatic, ART-naive adults from the United Kingdom and China [36], the antiviral inhibitory capacity of CD8^+^ T cells was highly predictive of CD4 cell loss in early HIV infection. Audige *et al.*[37] reported that fast progressors (those with a CD4 cell count <200 cells/μl within 7.5 years) had significantly lower postseroconversion CD4 cell counts than either intermediate (7.5–12 years) or slow (>12 years) progressors; fast progressors had cell-surface CD4 densities that decreased more rapidly than slow progressors.

## ANTIRETROVIRAL THERAPY DURING PRIMARY HIV INFECTION

There is global consensus that there is a favourable benefit : risk ratio for initiating ART in those with HIV-related symptoms or with a CD4 cell count less than 350 cells/μl. Because of the risk of disease progression in these individuals, the benefits of ART outweigh any potential risks of adverse drug reactions. Such a favourable benefit : risk ratio has not yet been established for initiating ART earlier in the course of infection in asymptomatic individuals.

Several recent publications provide further evidence that initiation of ART during primary infection may prevent the immunological deterioration, which would otherwise be seen in untreated HIV infection. In one observational study, 64% of individuals who initiated ART during primary infection maintained a CD4 cell count more than 900 cells/μl compared with only 34% of those who deferred ART to a later time [38^▪^]. In the Short Pulse Anti Retroviral Therapy at HIV Seroconversion (SPARTAC) trial, 366 adults with primary infection were randomized to receive either short-term (12 weeks) or longer term (48 weeks) immediate ART, or to defer ART until the CD4 cell count dropped to less than 350 cells/μl [39^▪▪^]. Immediate use of ART reduced the chance of experiencing a CD4 cell count less than 350 cells/μl while the patient remained on ART, but not beyond the duration of treatment. Using data from the observational CASCADE collaboration, Zugna *et al.*[40^▪^] reported that although individuals initiating treatment within 12 months of seroconversion were more likely to interrupt therapy than those initiating treatment during chronic infection, rates of virological failure and treatment change were similar between the two groups.

Although these studies demonstrate that ART can prevent the deterioration of the immune system which would otherwise be seen without treatment, they do not address whether those initiating ART during primary infection experience any long-term clinical benefit (in terms of reduced morbidity or mortality) from this treatment, and thus whether allowing CD4 cell counts to fall to lower levels will result in any appreciable negative consequences over either the shortterm or longterm. Unfortunately, such information can only be obtained from clinical endpoint studies with the requirement for substantially larger sample sizes. The ongoing Strategic Timing of Anti-Retroviral Treatment (START) study [41] aims to address this question.

## CONCLUSION

Although the clinical, immunological, and virological course of untreated HIV infection is variable, few persons followed for more than 8–10 years remain without any evidence of disease progression. Variation in viral characteristics, host defence responses (likely explained by variation in host genetics), and environmental factors may all contribute to the variation in the natural course of HIV infection. A better understanding of the relative influence of these factors is emerging. This line of research has the potential to identify novel targets for intervention to prevent and treat HIV-infected persons.

## Acknowledgements

None.

### Conflicts of interest

JDL is a member of the Executive and Scientific Steering Committees for the INSIGHT Network which is currently conducting the START trial. CAS has provided statistical input to various study designs from the INSIGHT group. There are no relevant financial conflicts of interest.

## REFERENCES AND RECOMMENDED READING

Papers of particular interest, published within the annual period of review, have been highlighted as:

▪ of special interest
▪▪ of outstanding interest

Additional references related to this topic can also be found in the Current World Literature section in this issue (pp. 353–354).

## Figures and Tables

**Table 1:** Definitions of long-term nonprogressors used in recent studies

Author (reference)	Symptoms allowed	ART allowed	Period of follow-up	CD4 requirement	Additional requirements/comments	Reported prevalence
Madec *et al.* [3]	Asymptomatic	No ART	>8 years after first positive HIV test	All ≥500 cells/μl	Study includes a high proportion of known seroconverters	9.0%
Okulicz *et al.* [4]	No AIDS	No ART	>7 years after diagnosis	All ≥500 cells/μl	–	5.0%
	No AIDS	No ART	>10 years after diagnosis	All ≥500 cells/μl	–	2.0%
Grabar *et al.* [5]	Asymptomatic	No ART	>8 years after diagnosis	Nadir >500 cells/μl	At least three CD4 and HIV RNA assessments available in 5 years prior to 2005	22.3%
	Asymptomatic	No ART	>8 years after diagnosis	Nadir >600 cells/μl	As above	11.4%
	Asymptomatic	No ART	>8 years after diagnosis	Nadir >600 cells/μl	As above, and positive CD4 slope over 5 years prior to 2005	2.8%
Mandalia *et al.* [6^▪▪^]	Asymptomatic	No ART	>7 years after diagnosis	>450 cells/μl	Stable CD4 slope (≥0 cells/μl per year) over entire follow-up period	0.2%
Gaardbo *et al.* [7]	Not stated	No ART	>10 years after diagnosis	>350 cells/μl	Viral load >5000 copies/ml	*N* = 14, prevalence not stated
Ballana *et al.* [8]	Not stated	No ART	>10 years after diagnosis	All >500 cells/μl	Viral load <10 000 copies/ml	*N* = 155, prevalence not stated

ART, antiretroviral therapy.
